# Development of a Complex Intervention to Improve Adherence to Antidiabetic Medication in Older People Using an Anthropomorphic Virtual Assistant Software

**DOI:** 10.3389/fphar.2019.00680

**Published:** 2019-06-21

**Authors:** Isa Brito Félix, Mara Pereira Guerreiro, Afonso Cavaco, Ana Paula Cláudio, Anabela Mendes, João Balsa, Maria Beatriz Carmo, Nuno Pimenta, Adriana Henriques

**Affiliations:** ^1^Unidade de Investigação e Desenvolvimento em Enfermagem (UI&DE), Lisbon Nursing School, Lisbon, Portugal; ^2^Centro de Investigação Interdisciplinar Egas Moniz (CiiEM), Instituto Universitário Egas Moniz, Monte de Caparica, Portugal; ^3^iMed.ULisboa, Faculty of Pharmacy, University of Lisbon, Lisbon, Portugal; ^4^Biosystems & Integrative Sciences Institute (BioISI), Faculty of Sciences, University of Lisbon, Lisbon, Portugal; ^5^Sport Sciences School of Rio Maior, Polytechnic Institute of Santarém, Santarém, Portugal

**Keywords:** relational agent, older adult, type 2 diabetes, medication adherence, virtual assistant, behavior change wheel, intervention development, complex intervention

## Abstract

**Introduction:** Improving adherence to antidiabetic medication is crucial, resulting in improved health outcomes, cost reduction, and minimization of waste. A lack of underlying theory in existing interventions may explain the limited success in sustaining behavior change. This paper describes the development of a theory and evidence-based complex intervention to improve adherence to oral antidiabetics in older people *via* a software prototype with an anthropomorphic virtual assistant.

**Methods:** The Behavior Change Wheel (BCW) was used to develop a theoretical understanding of the change process, corresponding to the first phase of the Medical Research Council Framework for developing and evaluating complex interventions. At the BCW core is a model of human behavior (COM-B), which posits that human behavior (B) results from the interaction between capabilities (C), opportunities (O), and motivation (M). Literature-derived medication adherence determinants were mapped onto COM-B components. Then, intervention functions (IFs) were selected employing the APEASE criteria. Finally, standardized behavior change techniques (BCTs) were chosen based on their suitability and their effectiveness on medication adherence trials. The prototype was developed for android devices; its core was implemented in Unity3D, using a female 3D virtual assistant, named Vitória.

**Results:** Two COM-B components were identified as main targets for behavior change—psychological capability and reflective motivation; these were linked with four IFs—education, persuasion, enablement, and environmental restructuring. Eleven BCTs were, in turn, linked with the IFs. An example of a BCT is “problem solving”; it requires users to pinpoint factors influencing non-adherence and subsequently offers strategies to achieve the desired behavior. BCTs were operationalized into the dialogues with Vitória and into supplementary software features. Vitória communicates with users verbally and non-verbally, expressing emotions. Input options consist of buttons or recording values, such as medication taken.

**Conclusion:** The present approach enabled us to derive the most appropriate BCTs for our intervention. The use of an explicit bundle of BCTs, often overlooked in interventions promoting medication adherence, is expected to maximize effectiveness and facilitates replication. The first prototype is being refined with users and health professionals’ contributions. Future work includes subjecting the prototype to usability tests and a feasibility trial.

## Introduction

Globally, diabetes affects more than 425 million people, of which one-third is older than 65 years (International Diabetes Federation, 2017). Type 2 diabetes (T2D) is the most prevalent form around the world, affecting 90% of the diabetic adults (Zheng et al., 2018). Glycemic control, which is key to prevent complications, requires sustained adherence to an appropriate diet, adequate physical activity, and, frequently, antidiabetic medication.

A systematic review including 27 studies found that adherence to antidiabetic medication can be as low as 38.5% (Krass et al., 2015). A recent study reported an overall antidiabetics adherence in older people of 72.4% (Juste et al., 2018). Additionally, Spanish elderly patients receiving oral antidiabetics showed rates of discontinuation for oral antidiabetics of 46.8% (Menditto et al., 2018).

Poor medication adherence in T2D is associated with negative outcomes, including increased morbidity and mortality, increased costs of ambulatory and hospital care, as well as loss of quality of life (Iuga and McGuire, 2014).

A wide array of factors associated with non-adherence has been described, which may differ according to study designs. For example, key nonpatient factors (e.g., healthcare systems limitations), non-modifiable patient factors (e.g., young age, low income level), and modifiable patient factors (e.g., beliefs about perceived treatment efficacy and medication burden) may be associated with non-adherence (Polonsky and Henry, 2016). Capoccia et al. (2016) mentioned lower adherence when medications were not tolerated or were taken more than twice daily.

Strategies to overcome poor medication adherence should consider the reduction of the medication burden, not only from a drug perspective (e.g., using fixed combinations) but also tools for an easier therapy management, plus responding to negative medication beliefs (Polonsky and Henry, 2016; Capoccia et al., 2016). Modifiable psychosocial factors belong to the overarching domains of knowledge, beliefs (and related cognitive constructs), behavioral skills, and coping (Gonzalez et al., 2016); thus, interventions targeting these aspects are expected to improve antidiabetic medication adherence.

Until recently, very few interventions to improve medication adherence were technology-based. Williams et al. (2014) reported a systematic review on effective interventions to improve medication adherence in T2D, in which the literature was searched from 2000 to 2013. The authors only found five studies using mobile phones or the Internet.

The advantages of technology-based interventions include automation and convenience of access. While simple electronic reminders, such as SMS, have shown improvement in medication adherence in chronic diseases (Vervloet et al., 2012), they are not without caveats. These reminders target essentially unintentional non-adherence and their long-term effects are uncertain (Vervloet et al., 2012). Technological development brought supplementary approaches, such as web-based interfaces, mobile applications, or smartwatches.

Currently, one of the most used technologies is referred to as mHealth apps (mobile health applications, typically for iOS or android devices). The rapid growth of these apps occurs in the context of aging population. The mHealth advances align well with the growing interest of older adults to integrate technology in the self-management of chronic conditions (Gilbert et al., 2015; Kim and Lee, 2017).

Concerning medication adherence, Anglada-Martinez et al. (2015) concluded that 65% of the mHealth application trials reported positive results. Virtual humans, designed to build long-term socioemotional relationships with users, can be incorporated in mHealth applications. Such virtual humans are commonly referred to as relational agents, although the term is often used interchangeably with virtual agent or assistant. There is a considerable body of knowledge on the effect or acceptability of relational agents in different populations. The acceptability to older people, including those with low literacy, has been studied (Bickmore et al., 2010). These authors found that individuals with inadequate literacy generally report higher levels of satisfaction with the relational agent and ask more questions than those with adequate health literacy. This has implications for our project, as the health literacy of older people in Portugal is limited (Mota-Pinto et al., 2010; Paiva et al., 2017).

Additionally, research suggests that a relational agent software is effective in promoting medication adherence and accepted by people with schizophrenia (Bickmore et al., 2010). Recently, the effect of an embodied conversional agent on antiretroviral therapy adherence was researched, using a 3-month pre-post design (Dworkin et al., 2019). This study demonstrated a positive effect on pill count, which improved from 62% at baseline to 88% at follow-up. The use of these agents to support diabetes self-care, including medication adherence, seems to have received less attention. Exceptions are papers describing coaching interventions for diabetes patients, which focus on medication adherence (op den Akker et al., 2011; Monkaresi et al., 2013). However, these papers lack data on usability or the effect on endpoints of interest.

The present paper aims to describe the development of a theory and evidence-based complex intervention to improve adherence to oral antidiabetics in older people *via* a software prototype with an anthropomorphic virtual assistant (VASelfCare). This is part of a wider intervention, targeting also lifestyle change of elderly T2D patients.

## Materials and Methods

Complex interventions include several interacting components that impact on the length and complexity of the causal chain and are influenced by features of the local context (Craig et al., 2008). The components usually include behaviors, characteristics of behavior (e.g., timing) and methods of organizing, and delivering those behaviors (e.g., setting and location). Many healthcare interventions are considered complex. The Medical Research Council (MRC) framework for developing and evaluating complex interventions has been widely employed (Bleijenberg et al., 2018); it provides guidance on the methodological and practical decision-making on healthcare interventions. The rationale for resorting to this framework is that interventions carefully designed and tested in an early stage are more likely to prevent negative or inconclusive trials (Craig et al., 2008).

**Figure 1:**
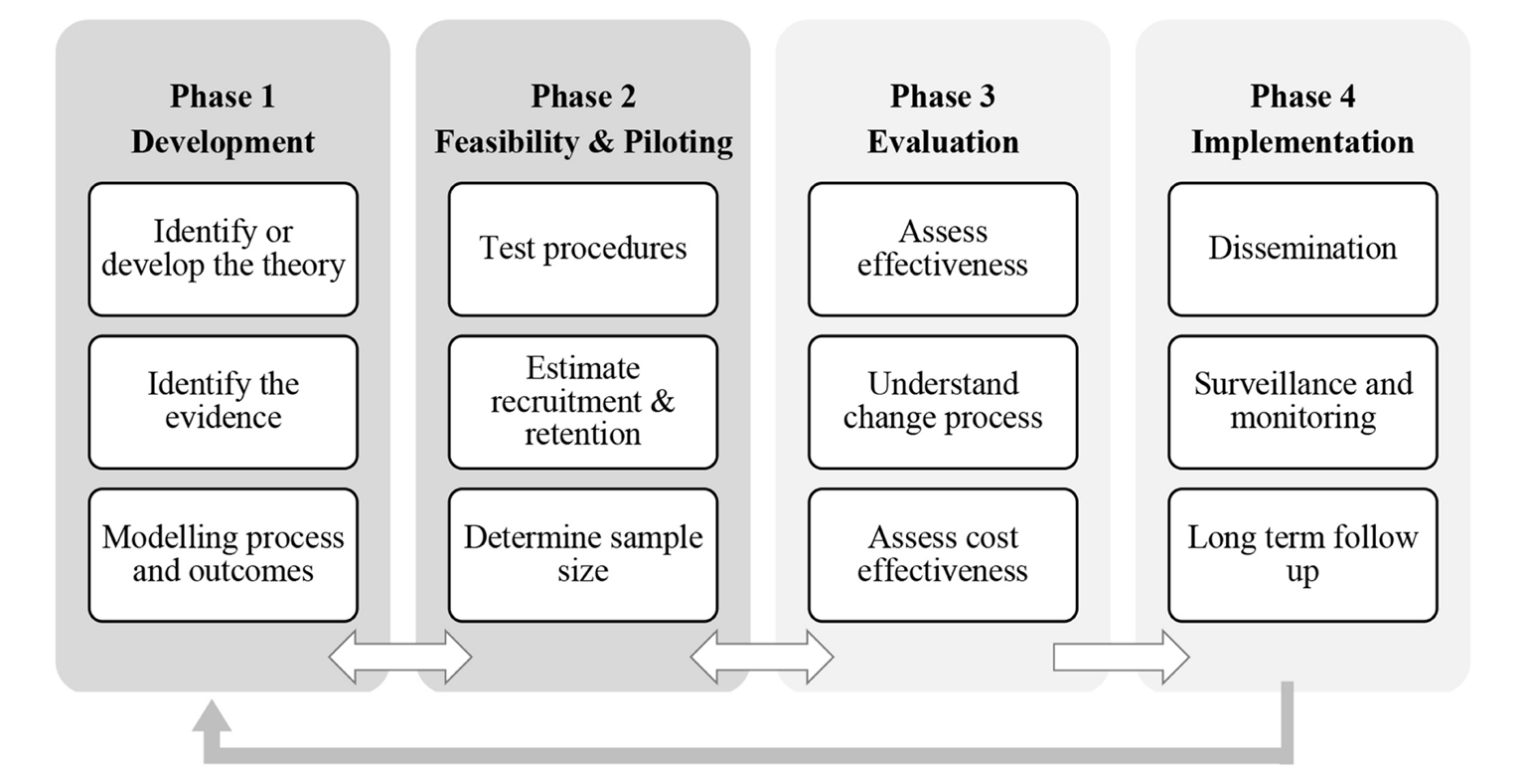
Medical Research Council framework (Craig et al., 2008).

**Figure 1** depicts the MRC framework phases. Phase 1 encompasses three elements (Craig et al., 2008), two of which are focused in the present paper: “identifying the existing evidence” and “identifying or developing theory.” Identifying the existing evidence included a mapping review on the effectiveness of relational agents, on the design of these interventions and on successful medication adherence interventions. We followed guidelines for self-management of people with T2D, focusing on key dimensions of lifestyle (diet and physical activity) plus medication adherence (Powers et al., 2015). It has been suggested that interventions targeting behaviors in a stepwise approach are more effective in promoting change than targeting multi-behaviors simultaneously (Sweet and Fortier, 2010).

The mapping review informed the intervention components. One of the intervention components is the nurse interaction with older people upon referral from the software prototype, in predefined circumstances. Moreover, the experience of Bleijenberg and colleagues (2018) with the MRC framework indicates that it is important to consider the elements of context, e.g., how the receiver (older people with T2D) and the provider (nurse) interact within the intervention. In Portugal, diabetes nursing consultations in primary care have been formally established as supplementary to medical consultations.

Another key task in phase 1 (**Figure 1**) is to develop a theoretical understanding of the underlying processes of change. The section Deriving the Active Ingredients of the Intervention describes the use of theory for deriving the active ingredients of the intervention. Then the section Development of the VASelfCare Software Prototype provides a brief description of how the software prototype was developed.

### Deriving the Active Ingredients of the Intervention

We chose the Behavior Change Wheel (BCW) (Michie et al., 2014) as the theoretical framework underpinning the intervention. The BCW is a recent approach to design research of behavior change interventions through a systematic and structured process (Michie et al., 2011; Cane et al., 2012). This process enables the evidence-based selection of intervention components, ensuring that the intervention targets the underlying determinants of behavior.

At the BCW core is a model of human behavior—the COM-B—which posits that human behavior (B) results from the interaction between physical and psychological capabilities (C), opportunities provided by the physical and social environment (O) and reflective and automatic motivation (M) (Michie et al., 2014). The COM-B is supported by the Theoretical Domains Framework (TDF), which describes 14 factors derived from 33 theories of behavior change. These factors fall under the categories of capability, opportunity, and motivation (Cane et al., 2012).

In the next sections, we will describe the stepwise approach to derive behavior change techniques (BCTs) for improving medication adherence based on the BCW.

#### Stage 1: Understanding the Behavior

We tackled this stage by reviewing the evidence on adherence and its determinants, which includes both barriers and facilitators to medication use. Several recent systematic literature reviews on determinants of adherence have been published. In particular, we draw on the seminal WHO on adherence determinants (Sabaté, 2003) and on systematic reviews and literature with the same scope to understand influences to medication use (Gellad et al., 2011; Kardas et al., 2013; Zeber et al., 2013; Capoccia et al., 2016; Yap et al., 2016; Choi and Smaldone, 2018). Subsequently, we mapped these determinants into the COM-B and identified key domains more influential to behavior change, through a multidisciplinary consensus discussion within the research team. This team has expertise in nursing, medication adherence, clinical pharmacology, and clinical communication.

#### Stage 2: Identifying Intervention Functions

Intervention functions (IFs) are defined as “broad categories of means by which an intervention can change behavior” (Michie et al., 2014, 192). Based on a systematic review of frameworks of behavior change interventions, Michie et al. (2011) put forward nine IFs. The BCW links COM-B domains to IFs and subsequent policy categories most likely to achieve behavioral change (Michie et al., 2011, 2014). Policy categories represent “types of decisions made by authorities that help to support and enact the intervention” (Michie et al., 2014, 235). We did not address these categories as they fell outside the scope of our intervention.

The IFs and their relation to the COM-B domains were organized in a mapping matrix (Michie et al., 2014). The former were selected based on their links to COM-B domains, as prescribed by the authors (Michie et al., 2014). The same IF can be linked to different COM-B domains. IFs most likely to affect behavior change were selected in a multidisciplinary consensus discussion within the research team, using the APEASE criteria: 1) affordability, 2) practicality, 3) effectiveness and cost-effectiveness, 4) acceptability, 5) side-effects/safety, and 6) equity (Michie et al., 2014).

#### Stage 3: Identifying Behavior Change Techniques

A BCT is an observable, replicable, and irreducible component of an intervention designed to change behavior (Michie et al., 2013). BCTs are known as the active ingredients of the intervention. They have been organized in a taxonomy, designated in brief by BCTTv1 (Michie et al., 2013; Cane et al., 2015), which provides a definition for each of the 93 techniques, enabling a consistent application.

There is guidance on the BCTs better suited to certain IFs and their underlying theoretical domains (Michie et al., 2008; Cane et al., 2015). From the list of 93 BCTs, the most appropriate for our intervention were selected based on:

- The most common BCTs under each IF (Michie et al., 2014).
- Effective medication adherence interventions from which BCTs could be derived (Nieuwlaat et al., 2014; Williams et al., 2014; Sapkota et al., 2015).
- A multidisciplinary discussion informed by the APEASE criteria, in particular on the practicality of including the BCT in light of the automated nature of the intervention.

### Development of the VASelfCare Software Prototype

We decided that the application would run in tablets with android system without requiring Internet connection. This decision was grounded on the need to secure inclusive access, considering that presently a significant percentage of older adults’ homes do not have Internet available (INE, 2014). Such decision, in turn, influenced software development options.

The overall development of the application was guided by usability principles for older people with T2D (Arnhold et al., 2014). For example, redundancy of both audio and written information may help reduce any communication shortcomings, such as lower eyesight accuracy and hearing deficits.

The core of VASelfCare solution was implemented in Unity3D (https://unity3d.com/pt), a software for the development of video games.

The virtual assistant chosen for this first prototype was a female 3D model with a realistic look, obtained from Daz3D (https://www.daz3d.com/). The choice for a 3D character is supported by studies that concluded that realistic-looking virtual agents are more appropriate for medical tasks (Ring et al., 2014; van Wissen et al., 2016). Other applications have also employed female figures as relational agents, for example (Bickmore et al., 2005a; Bickmore et al., 2005b).

Body animations and facial expressions are activated by the software that controls the virtual assistant behavior and verifies the context of the on-going interaction. Conveying emotions to users, beyond messages content, intends to establish an affective and effective communication. Furthermore, this has been acknowledged as the cornerstone for engagement and long-term use (Bickmore et al., 2010).

To support the virtual assistant speech, first dialogues had to be created, then the corresponding sound is generated and mouth animations that simulate the articulation of the sound were provided.

Dialogues were iteratively created by team members with a nursing and pharmacy background. Quality of the output was ensured by agreeing on principles at the outset of the process, by discussing dialogue structured regularly and through independent double-checking of scripts. To streamline this creative process, we adopted Yarn (https://github.com/InfiniteAmmoInc/Yarn), a graphical dialogue editor simple to use and compatible with the other software tools that were being used. Yarn provides an interface where the speech lines of the virtual assistant and the options that the user may choose (i.e., buttons in the interface) are inserted in text boxes. These are sequentially connected in a way that expresses the possible progressions of the dialogue during the interaction between the user and the virtual assistant.

General principles were deployed to write a comprehensive and optimal dialogue for each individual user (Migneault et al., 2006). One principle was tailoring content, which has been defined as “any combination of information and behavior change strategies intended to reach one specific person based on characteristics that are unique to that person related to the outcome of interest and derived from an individual assessment” (Kreuter et al., 2000). A meta-analysis showed that tailored web-based interventions have a significantly greater effect in health behavior outcomes than non-tailored counterparts (Lustria et al., 2013). Another principle was personalization, which broadly involves giving an indication that the software “knows who is talking to” (Migneault et al., 2006). A non-judgmental approach was employed to create the dialogues, resorting to a helpful-cooperative communication style (Niess and Diefenbach, 2016). Such an approach aims at creating rapport and a trust-bond, so that effectiveness of BCTs may be enhanced, along with other psychological benefits, as reported in the literature on virtual assistant-based interventions (Velicer et al., 2015).

A rule-based component was implemented to control the dialogue flow of the interaction. This component corresponds to the definition of a set of if–then rules (rules of the form “if *some conditions hold* then *execute some action*”), where the *conditions* may include context information regarding the interaction (e.g., user characteristics, such as age, or the date when interaction takes place) and the *action* represents the subsequent locutionary act performed by the virtual assistant. This approach allows greater flexibility in the dialogue definition and in the generation of diversified interactions.

Speech2Go from Harpo (https://harposoftware.com) was used to generate the audio files. This is a text-to-speech (TTS) software, which converts the written dialogues to audio speech. Since the virtual assistant is currently represented by a female character, the female voice of Inês, a European Portuguese voice, has been used. The speech rate has been slowed down, taking into consideration the target population.

Visemes describe the animations of the virtual assistant’s mouth that simulate phonemes’ articulation. Conversion of written dialogues to the viseme files is performed by a software developed in a previous project (Cláudio et al., 2015).

SQLite (https://www.sqlite.org/index.html) was the database engine chosen to control all the information stored locally in each tablet as it encompasses a secure storage mechanism. The local database, initially empty, will store clinical data registered by the care nurse, such as the prescribed antidiabetic medication, weight, and HbA1c. It will then be filled with data inserted during user interactions, including clinical data such as blood glucose levels, medication taken, and number of daily steps. These data are deployed in future interactions with the user and enable analysis at a later stage, contributing to the further development of the software prototype. Periodically, the database in each tablet is copied to an institutional computer using a secure connection and the access to this repository is reserved for credentialed personal. Privacy is further ensured by restricting access to this repository only to the researcher responsible for data analysis; data will be extracted omitting any reference that could identify the user.

## Results

### Deriving the Active Ingredients of the Intervention

#### Stage 1: Understanding the Behavior

The target behavior of this intervention is medication adherence, defined briefly as the behavioral response to an agreed medical recommendation (Sabaté, 2003). Recently, medication adherence has been defined as a process consisting of three stages: initiation, implementation, and discontinuation (Vrijens et al., 2012). The target behavior for this intervention was further defined as taking between 80% and 100% of the prescribed antidiabetic doses. The 80% cut-off point is underpinned by previous research that stratified adherent and non-adherent individuals based on predicting subsequent hospital admission across prevalent chronic conditions, including diabetes (Karve et al., 2009). The clinical implications of non-adherence to antidiabetic agents have been discussed earlier in the Introduction section.

To be effective the software prototype should target modifiable determinants of adherence (Allemann et al., 2016). Therefore, determinants with a negative effect on adherence, amenable of change by the prototype or other intervention components, were extracted from the literature (Gellad et al., 2011; Kardas et al., 2013; Zeber et al., 2013; Capoccia et al., 2016; Yap et al., 2016; Choi and Smaldone, 2018). For example, medication cost is not directly modifiable by the software prototype, but can be addressed *via* referral to the primary care nurse. The same applies to suspected adverse drug reactions, which the prototype cannot manage on its own. **Table 1** depicts the determinants influential on behavior change mapped into key domains of the COM-B.

**Table 1:** Adherence determinants mapped into the COM-B model.

Adherence determinants	COM-B components
Comprehension of disease and treatment	Psychological capability
Cognitive functioning (e.g., memory)	
Perception of illness	Reflective motivation
Concerns about medication	
Beliefs about medication necessity	
Outcome expectancies about treatment	
Self-efficacy	

#### Stage 2: Identifying Intervention Functions

**Table 2** presents the IFs selected and the reasons for their inclusion or exclusion. Overall, two IFs—“modeling” and “training”—were excluded because they were deemed impracticable to be applied *via* the software.

**Table 2:** Links between the COM-B model and relevant intervention functions.

COM-B components	Intervention function	Intervention function definition	Reasons for inclusion/exclusion (APEASE criteria)	Included/excluded from the next stage
Psychological capability	Education	Increasing knowledge or understanding	Considered affordable, practical, potentially effective, potentially acceptable, safe, and equative.	Included
	Training	Imparting skills	Not practical to delivery through the software prototype.	Excluded
	Enablement	Increasing means/reducing barriers to increase capability (beyond education/training) or opportunity (beyond environmental restructuring)	Considered affordable, practical, potentially effective, acceptable, safe, and equative.	Included
	Environmental restructuring	Changing the physical or social context	Considered affordable, practical, potentially effective, potentially acceptable, safe, and equative.	Included
Reflective motivation	Education	Increasing knowledge or understanding	Considered affordable, practical, potentially effective, potentially acceptable, safe, and equative.	Included
	Persuasion	Using communication to induce positive or negative feelings or stimulate action	Considered affordable, practical, potentially effective, potentially acceptable, safe, and equative.	Included
	Modelling	Providing an example for people to aspire to or imitate	Not practical to delivery through the software prototype.	Excluded
	Education	Increasing knowledge or understanding	Considered affordable, practical, potentially effective, potentially acceptable, safe, and equative.	Included
	Persuasion	Using communication to induce positive or negative feelings or stimulate action	Considered affordable, practical, potentially effective, potentially acceptable, safe, and equative.	Included
	Modelling	Providing an example for people to aspire to or imitate	Not practical to delivery through the software prototype.	Excluded
	Enablement	Increasing means/reducing barriers to increase capability (beyond education/training) or opportunity (beyond environmental restructuring)	Considered affordable, practical, potentially effective, potentially acceptable, safe, and equative.	Included

#### Stage 3: Identifying Behavior Change Techniques

Eleven BCTs were selected to be included in the software prototype (**Table 3**).

**Figure 2:**
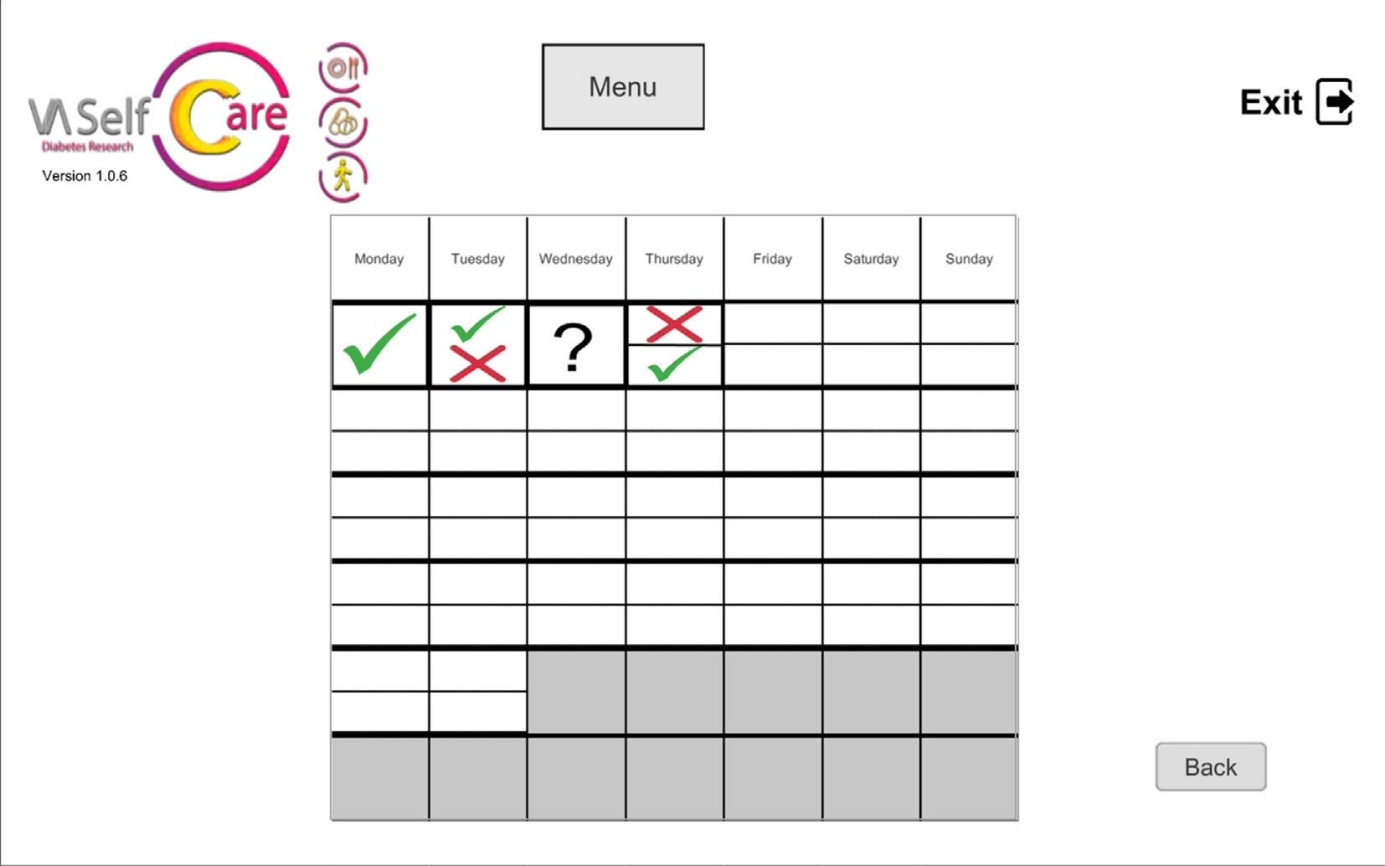
Calendar depicting antidiabetics-taking for one oral antidiabetic, two daily doses; question mark means no self-reported data (“feedback on behavior”).

**Figure 3:**
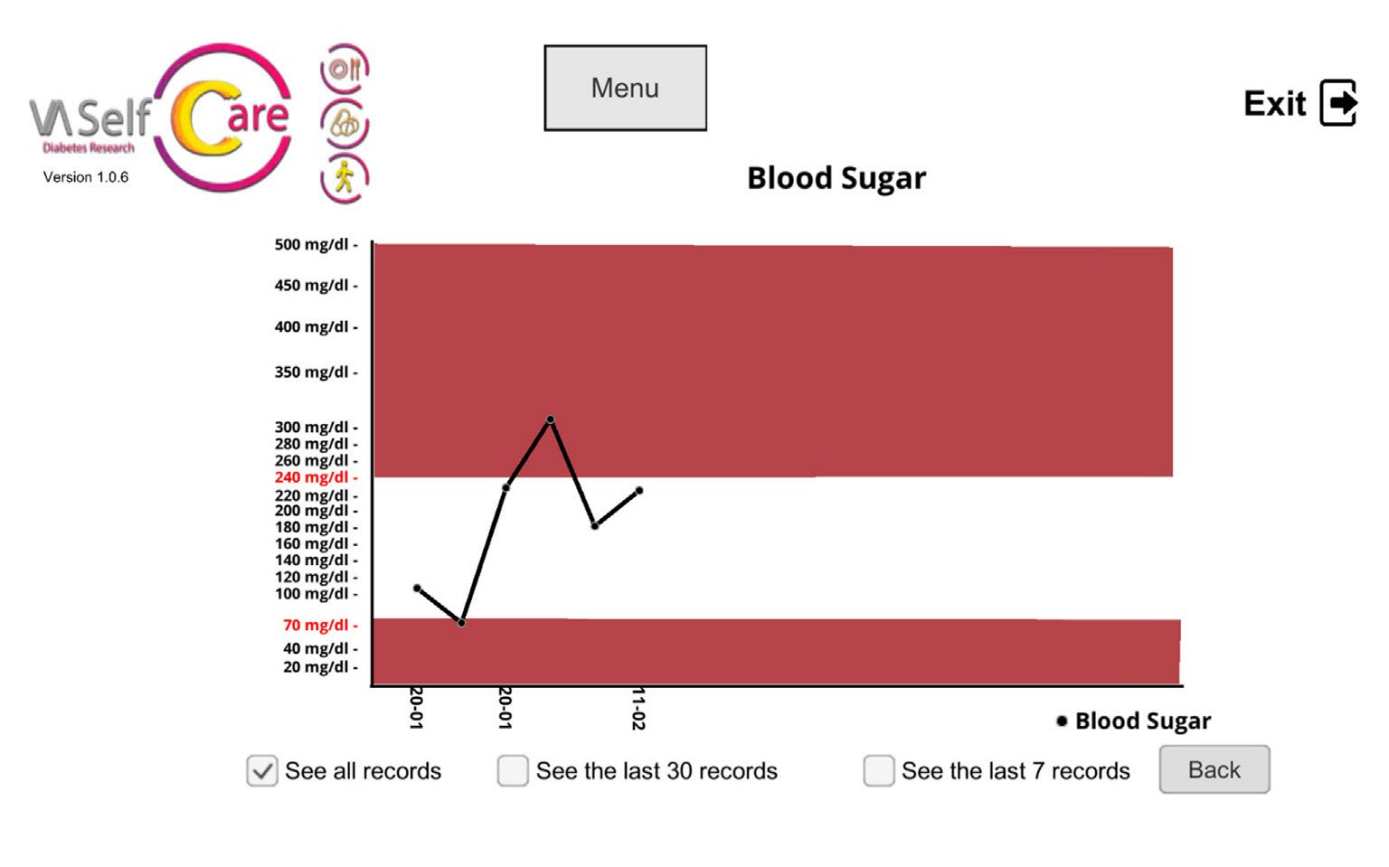
Chart to present blood glucose levels (“biofeedback”).

**Figure 4:**
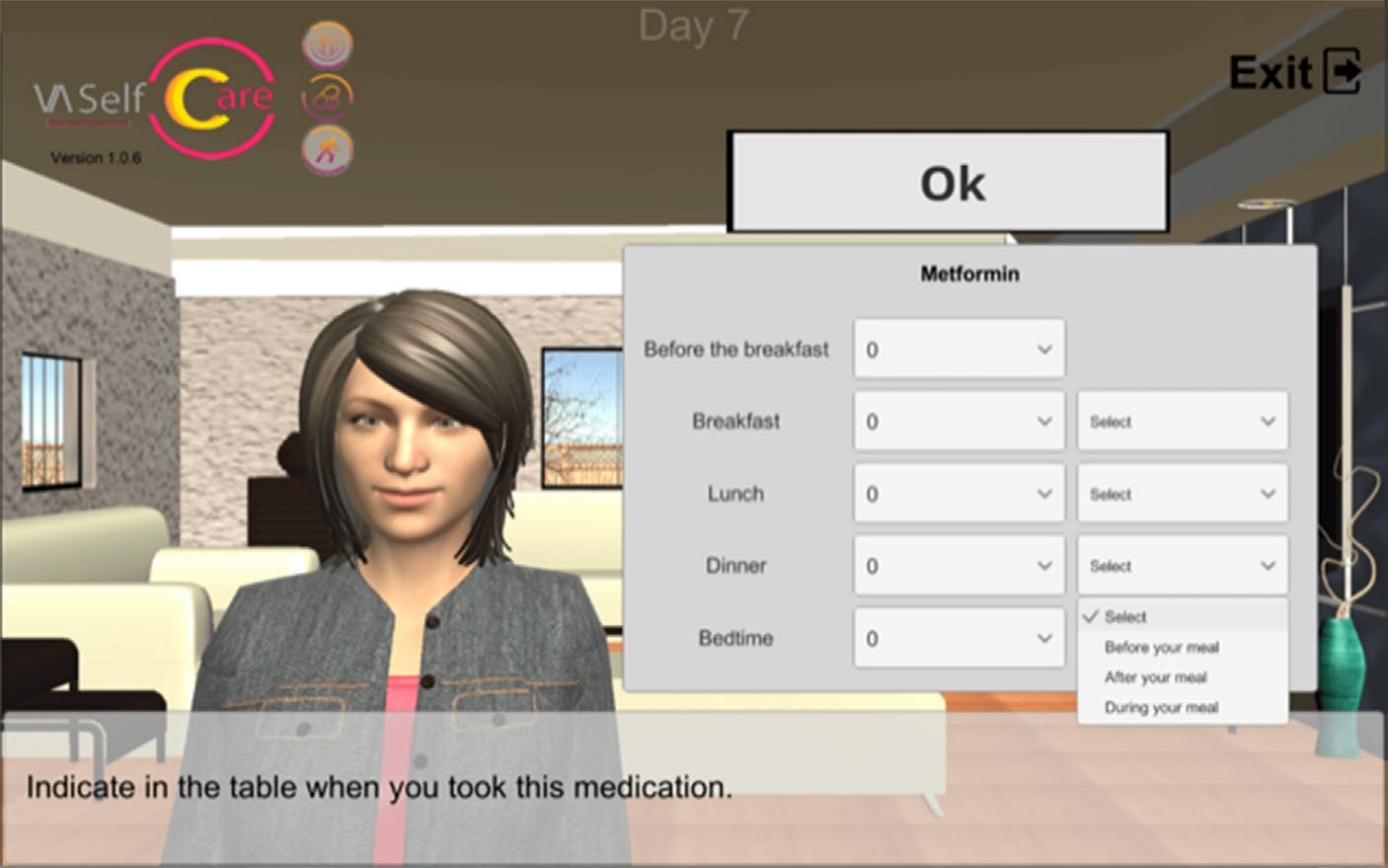
Virtual assistant collecting information on antidiabetics-taking (“self-monitoring of behavior”).

**Figure 5:**
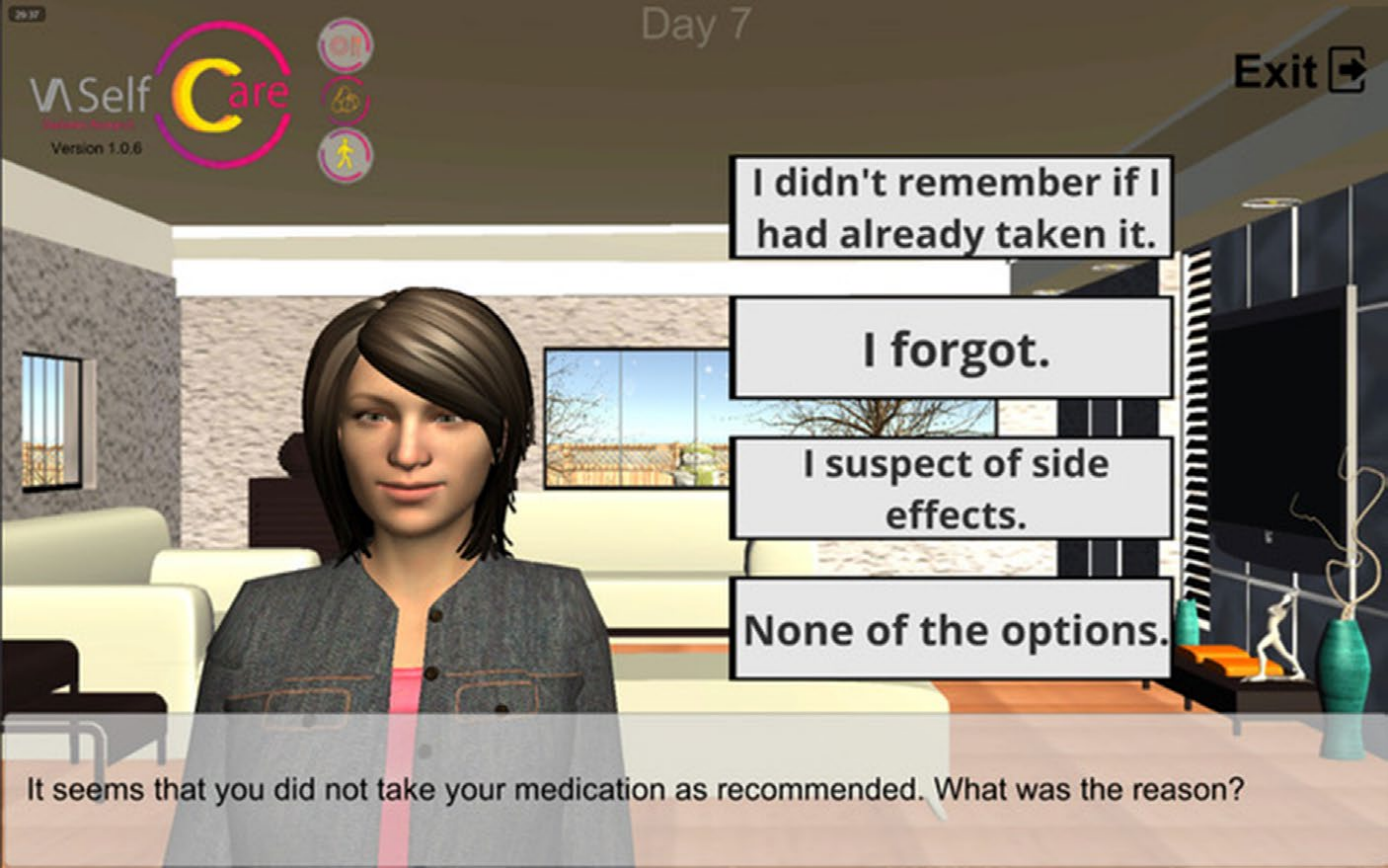
Virtual assistant listing factors that influence antidiabetics adherence (“problem-solving”).

The selected BCTs can serve more than one IF. For example, “feedback on behavior” could be linked to the “education” and “persuasion” functions. This BCT was operationalized by providing information on medication-taking behavior *via* a chart (**Figure 2**), described by the virtual assistant, plus giving supportive messages about the user’s abilities to perform the desired behavior. **Figures 3**, **4**, and **5** depict software interfaces representing “biofeedback,” “self-monitoring of behavior,” and “problem-solving,” respectively. While the software prototype is in Portuguese, we present figures in English to facilitate readers’ understanding.

**Table 3:** BCTs selected for the VASelfCare software prototype (antidiabetics adherence).

Intervention functions		BCTs	Definition (Michie et al., 2014)
**Education**	**Persuasion**	Feedback on behavior (2.2)	Monitor and provide informative or evaluative feedback on performance of the behavior (e.g., form, frequency, duration, intensity)
		Information about health consequences (5.1)	Provide information (e.g., written, verbal, visual) about health consequences of performing the behavior
		Biofeedback (2.6)	Provide feedback about the body (e.g., physiological or biochemical state) using an external monitoring device as part of a behavior change strategy
		Self-monitoring of behavior (2.3)	Establish a method for the person to monitor and record their behavior(s) as part of a behavior change strategy
		Prompts/cues (7.1)	Introduce or define environmental or social stimulus with the purpose of prompting or cueing the behavior. The prompt or cue would normally occur at the time or place of performance.
**Enablement**		Action planning (1.4)	Prompt detailed planning of performance of the behavior (must include at least one of context, frequency, duration, and intensity). Context may be environmental (physical or social) or internal (physical, emotional or cognitive) (includes “implementation intentions”)
		Social support (unspecified) (3.1)	Advise on, arrange or provide social support (e.g., from friends, relatives, colleagues, “buddies,” or staff) or non- contingent praise or reward for performance of the behavior. It includes encouragement and counseling, but only when it is directed at the behavior
		Social support (practical) (3.2)	Advise on, arrange, or provide practical help (e.g., from friends, relatives, colleagues, “buddies,” or staff) for performance of the behavior
		Goal setting (behavior) (1.1)	Set or agree on a goal defined in terms of the behavior to be achieved Note: only code goal-setting if there is sufficient evidence that goal set as part of intervention; if goal unspecified or a behavioral outcome, code 1.3, goal setting (outcome); if the goal defines a specific context, frequency, duration, or intensity for the behavior, also code 1.4, action planning
		Problem solving (1.2)	Analyze, or prompt the person to analyze, factors influencing the behavior and generate, or select strategies that include overcoming barriers and/or increasing facilitators (includes “relapse prevention” and “coping planning”)
**Environmental restructuring**		Restructuring the physical environmental (12.1)	Change, or advise to change the physical environment in order to facilitate performance of the wanted behavior or create barriers to the unwanted behavior (other than prompts/cues, rewards, and punishments)

### Development of the Medication Adherence Component of the VASelfCare Prototype

Prior to the user’s first interaction with the prototype, registration by a nurse providing care in diabetes consultations is required. There is a specific interface for this purpose that includes mandatory clinical information, such as the prescribed antidiabetic medication and the last HbA1c level.

When a user interacts with the prototype for the first time, a number of features are available. A key feature, described in the next section, is the dialogue view, in which the user interacts with Vitória, an anthropomorphic virtual assistant. Supplementary features are offered in other views. The prototype is designed to allow no more than one full interaction with Vitória per day. Access to the other views is unrestricted.

#### Interaction With the Dialogue View

Vitória is capable of speaking, by means of a synthetic voice, and features also text subtitles, reproducing the verbal content. Furthermore, Vitória expresses herself non-verbally with users, providing emotions through facial and body animations, depending on the user’s response. The user communicates with Vitória using buttons or by recording values, such as medication taken.

To attain a more convincing experience, the 3D scenario where Vitória is displayed changes, for example, according to the time of the day and season of the year.

Initially, the user is prompted to select a component to start with (medication adherence or lifestyle changes—diet or physical activity). Assuming the medication component is selected, the first 3 days are devoted to evaluation. The main purpose of this phase is to collect data on user’s knowledge about antidiabetic agents, current behavior on medication-taking and perceived self-efficacy in medication management, for future tailoring of the intervention.

The evaluation phase was structured as a six-step interaction: opening; social talk; assessment; feedback; pre-closing; and closing. This structure has been adapted from the literature (Bickmore et al., 2005b). The “opening” step consists of greeting the user and it is followed by the “social talk,” which includes inquiries about the user’s general emotional and physical state (e.g., “how are you feeling today?”). “Assessment” is the step in which questions about the aforementioned constructs are posed. In the “feedback” step, brief information on the answers is provided. Finally, the contents of the next interaction are described (“pre-closing”) and a farewell is delivered (“closing”).

The second phase, designated by follow-up, has a main purpose of promoting the desired behavior or to maintaining it. The dialogues in this phase are also structured in repeated sequential steps, described in the literature (Bickmore et al., 2005b). The first two (opening and social talk) and the last two steps (pre-closing and closing) correspond to those described in the evaluation phase. In the “review tasks” step, information is collected about previously agreed tasks or behaviors. Then, feedback is provided and behavior determinants are discussed where pertinent (“assess”). The “counseling” step has a twofold end. Firstly, it offers strategies to overcome previously identified adherence barriers, if applicable. Secondly, it provides tailored educational contents to users. This is exemplified by information about adverse drug reactions, according to knowledge gaps identified in the evaluation phase. In the “assign tasks” step, the user negotiate new behavioral goals and tasks with Vitória.

The selected BCTs are incorporated into different steps of the dialogues, as shown in **Table 4**.

**Table 4:** Description of the operationalization of each BCT.

BCTs	Strategies	
	Dialogue view	Other views
Feedback on behavior (2.2)	Verbal and visual information on antidiabetics-taking, *via* Vitória’s speech, using a helpful-cooperative communication style, and *via* a calendar (“assess” step).	Calendar depicting antidiabetics-taking (the same used by the virtual assistant)
Information about health consequences (5.1)	Verbal explanation on the consequences of not taking antidiabetic agents, *via* Vitória’s speech, using a helpful-cooperative communication style. (“counseling” step)	Not applicable
Biofeedback (2.6)	Not applicable	Charts with HbA1C levels on baseline, 3 and 6 months and with self-monitoring of blood glucose levels (if conducted)
Self-monitoring of behavior (2.3)	Information on antidiabetics-taking is collected by the virtual assistant *via* a self-completed form (“review tasks” step)	Record of antidiabetics-taking (available only if not asked by the virtual assistant; depends on the stage of the intervention)
Prompts/cues (7.1)	The virtual assistant offers the possibility of setting up a customizable medication reminder if forgetfulness is identified as a determinant of non-adherence (“counseling”)	Setting up a customizable medication reminder
Action planning (1.4)	The virtual assistant offers the possibility of setting up a plan, depending on the determinants of non-adherence (“counseling” step)	Not applicable
Social support (unspecified) (3.1)	The virtual assistant suggests resorting to a friend, relative or health professional, depending on the determinants of non-adherence (“counseling” step). Vitória’s helpful-cooperative communication style throughout several dialogue steps provides occasionally non-contingent encouragement.	Not applicable
Social support (practical) (3.2)	Referral to a health professional when non-adherence determinants cannot be addressed by the application (“counseling” step)	Not applicable
Goal setting (behavior) (1.1)	The virtual assistant asks whether the user intends to take oral antidiabetics as prescribed (“assign tasks” step)	Not applicable
Problem solving (1.2)	The virtual assistant lists the factors influencing antidiabetics adherence and generate strategies that include overcoming barriers or increasing facilitators (“assess” and “counseling” steps)	Not applicable
Restructuring the physical environmental (12.1)	The user is advised to change the physical environment according to the barriers that influence medication adherence (“counseling” step)	Not applicable

Tailoring was applied not only at the information level, but also in what concerns specific BCTs. For instance, social support (**Table 4**) is only employed when non-adherence determinants cannot be addressed by the software prototype.

## Discussion

This article illustrates a systematic, evidence-driven, and theory-based approach to specify the content and active ingredients of a software prototype, devised to improve medication adherence in older adults with T2D.

Older age has been related to better medication adherence; for example, a systematic review identified increasing age as a factor positively associated with adherence to diabetes medication (Krass et al., 2015). Likewise, a meta-analysis by Choi and Smaldone (2018) indicates that the elderly are more likely to take diabetes medication as prescribed, when compared with younger adults. While *per se* older age seems to affect positively adherence, it is widely recognized that non-adherence is multidimensional. Other factors such as multiple morbidities, regimen complexity, cognitive decline, dexterity, and medication cost can impair adherence (Gellad et al., 2011). This case is illustrated by recent Portuguese data on medication adherence in type 2 diabetic patients measured by the Morisky Medication Adherence Scale (MMAS-4), which categorizes adherence in good, median, and bad (Pinto et al., 2019). The 60 to 79 years group presented both a higher proportion of good and bad medication adherence in relation to lower age groups. The fact that adherence interventions in older people merit research efforts is illustrated by the plethora of trials addressing this population group (Marcum et al., 2017).

We departed from published literature on potential determinants of medication adherence to ensure that the intervention targets known modifiable factors. Four IFs associated with psychological capability and reflective motivation in the COM-B model were subsequently selected—education, persuasion, enablement, and environmental restructuring—resorting to structured criteria (APEASE). Finally, 11 standardized BCTs were chosen from the BCTTv1, based on their suitability to the selected IFs and their effectiveness on medication adherence research trials. This developmental process was guided by the work of Cane and colleagues (2015) and the mapping work from Michie and collaborators (2008).

Morrissey et al. (2016) surveyed 166 smartphone medication adherence applications on the market to identify the BCTs present. From a range of 93 possible techniques (Michie et al., 2013), only 12 were found in the evaluated applications. In accordance with our work, the most common ones were “action planning” and “prompt/cues,” followed by “self-monitoring” and “feedback on behavior” (Morrissey et al., 2016).

Interventions to improve medication adherence often use BCTs combinations. Future research could examine which particular BCTs or combinations of BCTs are most effective in changing the medication-taking behavior in older people *via* a relational agent software. A recent study based on a different BCTs taxonomy found that specific BCTs configurations were effective in promoting medication adherence in the context of non-digital interventions (Kahwati et al., 2016). Overall, there is paucity of evidence on this topic, in particular concerning BCTTv1, maybe related to its novelty.

From the software standpoint, we have obtained a first prototype of a multi-behavior intervention, designed in light of usability requirements of older adults. It features an anthropomorphic virtual assistant, designed as a relational agent, able to communicate verbally and non-verbally in an empathic fashion. The agent tailors information to users’ level of knowledge, self-reported adherence, and perceived self-efficacy in managing medication. Moreover, it personalizes the interaction by, for example, mentioning the user’s name and referring to a relative or friend. The prototype offers several features in addition to the dialogue with the virtual assistant, such as a calendar depicting antidiabetics-taking behavior and a chart with HbA1C levels.

The question of whether applications to promote medication adherence using anthropomorphic virtual agents are advantageous when compared to their simpler, text-only counterparts, remains essentially unanswered. The few studies published present limitations, such as relatively small and younger non-clinical populations, which raises generalizability issues for older populations with prevalent chronic diseases. For instance, one study compared an application for behavior change in healthy eating using a female virtual agent versus simple text messages in a sample of 60 higher education students (Mazzotta et al., 2009); information quality and persuasion strength were better rated for the virtual agent modality. By contrast, a study comparing a virtual coach for healthy lifestyle versus text messages in a sample of 43 office workers was unable to demonstrate the virtual agent superiority (op den Akker et al., 2016). Once our intervention is matured, it can get trialed, contributing to answer this question.

### Strengths and Limitations

One of the strong points of our work is the theory-driven approach we pursued to the development of the medication adherence intervention. Absence of a theoretical underpinning has been associated with limited effectiveness in these interventions in older people and other patient groups (Nieuwlaat et al., 2014; Patton et al., 2017). Resorting to theory enables a better understanding of the behavior change process (Patton et al., 2017) and tends to have a significant effect on outcomes (Webb et al., 2010).

Another strength is providing a detailed description of the chosen BCTs. Published work on the development of behavior change interventions does not always offer such a description (Wells et al., 2012; Glasziou et al., 2014), which is critical to tout reproducibility. Portraying the content used in an intervention is crucial for reporting, replication, and synthesizing evidence (Proctor et al., 2013; Hoffmann et al., 2014).

However, this work presents also limitations. One of the limitations is whether the behavior change strategies devised by us truly represents a particular BCT. For instance, we were of the understanding that monitoring and recording blood glucose levels did not represent “self-monitoring of outcomes of behavior (2.4)”, as we did not provide a method for that purpose, indicating timing and frequency, as others did (Goyal et al., 2016). We attempted to overcome this uncertainty by in-depth discussion within the research team, aided by examples of BCTs in the scientific literature.

Another drawback is the fact that evidence on the effect of BCTs in medication adherence is limited. For instance, while a Cochrane review reported that education, counseling, daily treatment support, and additional support from family or peers were effective for improving adherence (Nieuwlaat et al., 2014), the interventions description may be insufficient to allow coding with a taxonomy. Furthermore, there are cases in which no standardized description of the active components is provided, nor referenced to a taxonomy. This is exemplified by a systematic review on effective interventions to improve medication adherence in T2D (Williams et al., 2014). Problem-solving emerged as effective in improving medication adherence and HbA1c levels (Williams et al., 2014), but it is arguable what it actually means. In other studies interventions addressed problem-solving through action plans and goal setting (Walker et al., 2011; Bogner et al., 2012; Sapkota et al., 2015); it is unclear whether these techniques are identical to their counterparts in BCTTv1. Describing interventions using a comprehensive taxonomy with agreed definitions could promote a better integration of research findings into practice and consequently to building a cumulative knowledge of intervention effectiveness (Presseau et al., 2015).

From an operational standpoint, one of the limitations of the current prototype is that medication adherence and tolerability is assessed only in respect to oral antidiabetics. Future efforts should be directed to the full medication regimen of users and not only blood glucose lowering medication.

The virtual assistant was designed to establish rapport and trust and to provide automated, long-term support to older adults with T2D, supplementing nursing and other health consultations. As previously mentioned, the virtual assistant is able to discuss nonadherence determinants and, according to the adherence barrier identified, provides strategies to overcome them. For example, beliefs about medicines may be tackled by providing information about antidiabetics medication and the disease. This represents a step ahead of many currently available applications. However, it is unlikely that the relational agent is able to manage all instances of non-adherence. The prototype is programed to refer to the primary care nurse or other healthcare professional if problems persist, potentially preventing sustained lack of adherence.

### Future Work

We have obtained a first prototype of a software application with an anthropomorphic virtual assistant, which has been used to elicit informed opinions on pre-requisites from nine older people with T2D and 19 health professionals’ in five primary care units of the Portuguese national health service (ethical approval 6104/CES/2018/ARSLVT). Results were overall positive and highlighted opportunities for improvement. These were prioritized and actioned.

In parallel with the software development, we are conducting a cross-sectional study in three primary care units to characterize the study population in respect to variables of interest, such as functional and cognitive status, HbA1c levels, and medication adherence. These results will inform the feasibility trial.

The next step is subjecting the prototype to usability tests in a purposive sample of around 10 older patients with T2D, employing the Portuguese version of the System Usability Scale (SUS) (Martins et al., 2015). Usability will also be evaluated by up to five experts, chosen by their experience in aspects such as interface design and diabetes management. This iteration will contribute to further fine-tune the prototype.

Subsequently, the software prototype will be tested in a non-randomized, non-controlled feasibility trial in participating primary care units. For this trial, we envisage to recruit about 20 end users enrolled in nursing consultations. Inclusion and exclusion criteria will be informed by the aforementioned cross-sectional study. Acceptability to users will be researched by focus groups, conducted at the end of the feasibility trial. This represents the second phase of the MRC framework.

## Conclusions

The BCW enabled the identification of the most appropriate BCTs for a novel intervention to promote adherence to antidiabetic medication in older people *via* an anthropomorphic virtual assistant. This stepwise approach involved the identification of four IFs, linked to two key COM-B categories (psychological capability and reflective motivation). Subsequently, 11 BCTs were considered potentially effective. The process outlined here can be used by researchers to guide a comprehensive intervention development, maximizing effectiveness, and facilitating replication.

## Author Contributions

IF, MG, and AH conceived the idea for the paper. IF guided the team through the behavior change wheel theoretical framework. All authors contributed to the first draft of the manuscript. IF and MG performed a first critical review, which was then commented by all authors. All authors approved the manuscript in its final version for submission and agreed to be accountable for the work presented.

## Funding

This project is supported by FCT and Compete 2020 (grant number LISBOA-01-0145-FEDER-024250, 02/SAICT/2016). It is also supported by UID/MULTI/04046/2019 Research Unit grant from FCT, Portugal (to BioISI).

## Conflict of Interest Statement

The authors declare that the research was conducted in the absence of any commercial or financial relationships that could be construed as a potential conflict of interest.
